# How Can Eastern/Southern Mediterranean Countries Resolve Quality and Safety Issues in Transfusion Medicine?

**DOI:** 10.3389/fmed.2018.00045

**Published:** 2018-02-27

**Authors:** Antoine Haddad, Tarek Bou Assi, Olivier Garraud

**Affiliations:** ^1^Department of Clinical Pathology and Blood Banking, Sacré-Coeur Hospital, Lebanese University, Beirut, Lebanon; ^2^EA3064, Faculty of Medicine of Saint-Etienne, University of Lyon, Saint-Etienne, France; ^3^Department of Laboratory Medicine, Psychiatric Hospital of the Cross, Jal El Dib, Lebanon; ^4^Department of Laboratory Medicine and Blood Banking, Saint Joseph Hospital, Dora, Lebanon; ^5^Institut National de la Transfusion Sanguine, Paris, France

**Keywords:** transfusion, Southern Mediterranean, Eastern Mediterranean, quality, safety, VNRD, blood supply

## Abstract

Unlike their Western counterparts, some of the Eastern/Southern Mediterranean countries lack centralized coordinated blood transfusion services leading to an unequal blood safety level. This was recently highlighted by a recent World Health Organization (WHO) regional committee report in which WHO urges these countries to establish and implement a national blood system with well-coordinated blood transfusion activities and to make attempts to reach 100% voluntary non-remunerated blood donation. The objective is thus to meet the same levels or standards as Western countries in term of self-sufficiency and blood safety. This raises the question whether these countries can either comply with Western countries’ guidelines and experiences or develop their own safety scheme based on proper sociopolitical and economic features. Another option is to identify efficient and cost-effective strategies setup successfully in neighbor countries sharing cultural and economic features. To address this issue—and make an attempt to achieve this goal—we designed a number of surveys specifically addressed to Mediterranean countries, which were sent out to the national authorities; so far, five surveys aim at covering all aspects in blood collection, processing, testing, inventory and distribution, as well as patient immune-hematological testing and follow-up (including surveillance and vigilances). It is anticipated that such practice can help identifying and then sharing the more successful and cost-effective experiences, and be really focused on Mediterranean areas while not necessarily copying and pasting experiences designed for Western/Northern areas with significantly distinct situations.

## Introduction

Despite having lower to upper middle-income economies, Eastern/Southern Mediterranean countries, compared with high-income countries (often referred to as either Western or Northern countries), provide similar transfusion therapies for a wide range of diseases and conditions. This comprises, among others, care for thalassemia major, and to a lesser extent, sickle cell disease patients, and assistance to transplantation programs, including stem cells (1, 2). However, unlike their Western counterparts, some of these countries lack centralized or coordinated blood transfusion services resulting in different levels of blood safety in terms of quantity and quality: insufficient blood supply, unequal availability of blood components (BCs) and nationwide health-care coverage, inadequate financial and human resources, etc. (3, 4). All of which were regularly highlighted by World Health Organization (WHO) reports, urging these countries to establish and implement a national blood system with well-coordinated blood transfusion activities. In parallel to its recommendation on blood use and surveillance, WHO also advised all countries to achieve a 100% voluntary non-remunerated blood donation (VNRBD) objective by the year 2020 (recently postponed to 2025 for Eastern Mediterranean countries) (5, 6). In stating this, WHO considers that the fundamental strategy to ensure timely access to safe and sufficient supplies of blood and blood products is the development of a nationally coordinated blood transfusion service based on VNRBDs without any other alternative (7).

## Current Questions Addressed by Developing Countries Regarding their Blood Collection and Transfusion Programs

Almost all countries in the process of implementing or strengthening their own national blood transfusion program must address seven key questions:

– (ai) How can we meet the clinical demand and become self-sufficient in procuring BCs?
– (aii) How can we guarantee donors’ safety during blood collection?
– (aiii) How can we ensure BCs are issued at the safest level (at least regarding transfusion-transmitted infections and immunohematological compatibility)?
– (aiv) How can we ensure a quality management-driven org-anization?
– (av) How can we set up a surveillance system to follow-up donors, the BC chain process and recipients?
– (avi) How can we guarantee optimal clinical use of blood and ensure Patient Blood Management (PBM) while avoiding inadequate transfusions and/or loss of expired blood products?
– (avii) How can we educate all staff categories (and/or other stakeholders)?

It is evident that not all countries are at the same level of progress toward these goals. In fact, the majority face a number of obstacles such as (8):

– (bi) Available financial and human resources (including educational level).
– (bii) Cultural habits, traditions, and experiences (including perception of blood donation or infusion and quality management).
– (biii) Unfavorable epidemiological conditions (active circulation of vectors, viruses, and other pathogens that may be transmitted by blood).
– (biv) Multiethnic population with antigenic diversity that makes immunohematological matching difficult to achieve for some patients.

These goals cannot be achieved without a strong commitment from the public authorities and the backing of the Ministry of Health in the country concerned.

Bearing in mind that quality, safety, education, surveillance, and vigilance program apply to all three aspects, the blood transfusion process can commonly be reported as a three-legged stool, i.e., the “A, B, C” of the process:

- Donors, donations, and/or blood collection.
- BCs (i.e., blood outside the donor and not yet transfused to the recipient).
- Recipients.

## The “A, B, C” of the Blood Collection and Transfusion Process with Special Reference to Developing Countries

### Donors/Donations/Blood Collection

It would be tempting to start with the “A” leg of the stool; however, it must be borne in mind that blood donation and/or collection only exist because there is a demand. There are three major approaches for analyzing this demand: (i) a passive analysis, which is the easiest and consists in retrospectively reviewing all BCs issued over a defined period of time for predefined clinical situations (obstetrical bleeding, trauma, malaria, etc.); (ii) an active or prospective analysis, more difficult to address, which consists in predicting the needs in a given population to fulfill certain clinical indications such as cancer therapy, transplantation, and internal medicine; (iii) a combined (active and passive) analysis, which simultaneously reviews historical data and prospects the needs based on the development of the patient recruitment processes. Therefore, it is instrumental to stratify the actual needs and anticipate any increase in blood demand based on the available hospital strategies to recruit patients and on the consensuses regarding transfusion strategies for every patient category. All of the above is aimed at defining whether a system is self-sufficient or not. Indeed, can anyone consider a system self-sufficient when it only ensures BCs on a daily basis (emergency or/and bleeding situations) as regularly seen in developing countries? Consequently, it can be deduced that self-sufficiency is difficult to define and should be driven by audits assessing both short (or daily basis) and long-term needs.

Once the demand is defined, blood collection programs can be launched to build up inventories. Here again, there are essentially two main pathways: the first applies to small Hospital Blood Banks (HBBs) and consists in fulfilling the arbitrary need on a daily basis. The second—usually seen in larger settings—is based on a program, adjusted according to the statistical consumption of blood. The latter is more suitable with the VNRD-based blood supply system where mobile drives in partnership with non-governmental organizations (NGOs) such as the Red Cross/Red Crescent, can eventually be planned in advance.

The respective values of VNRD and replacement donation can now be discussed.

Replacement donation consists in donating blood voluntarily in case a relative is in need, therefore contributing to blood bank replenishment. This donation mode is predominant in almost all Mediterranean countries. In fact, a recent Greek study found that donors seem to be more sensitized by the need in BCs rather than altruism, contrary to what is seen in VNRD systems (9). The donation is addressed specifically to the bank and not to the patient, since the donor and recipient are not required to have an identical blood group. The recipient will be transfused with already processed BCs, bearing in mind that the anonymity process is always guaranteed. No direct benefits are provided by the patient, donor or blood bank. However, indirect benefits cannot be ruled out for each party. Based on the literature, replacement donation should not be abandoned since it is more efficient in small facilities where the collection of blood and inventory replenishment occurs at all times simultaneously with the BC delivery activity (10).

However, both systems have their own advantages and disadvantages (11). The VNRD system clearly favors intergroup solidarity (region and nation), while the replacement system favors intragroup solidarity (village or neighborhood, family, and work station). Complying with WHO recommendations and abandoning replacement donation should, if adopted, be scheduled progressively and strongly encouraged and supported by the national authorities.

The following two examples appear to illustrate this statement: (a) the first is the Lebanese experience (12). In this country, blood banking does indeed largely depend on replacement donors for several reasons: decentralized system, predominance of the private health-care sector over the public (i.e., a fragmented system) and the cultural habits of its inhabitants who are used to react in emergency situations (13). Recently, NGOs started taking initiatives to promote national solidarity and VNRD and have made considerable progress (14). However, this should be further encouraged by the national authorities who lack complete involvement (12) due to political issues. Meanwhile, should Lebanon encourage family replacement donors who meet all the classical criteria of VNRD to donate regularly? In fact, some authors consider these donors legitimate and indispensable (10, 15). (b) The second experience is the Moroccan one where VNRD is highly valued and where national authorities are fully involved under the blessing of the Royal Family. In fact, the Royal Family has been photographed while donating blood and their pictures are displayed in blood centers clearly to motivate donors and promote voluntary blood donation. Despite cultural similarities with Lebanon, Morocco, which is a centralized state, seems to be on the right track toward achieving 100% VNRD (the WHO target).

VNRD is recognized as the universal goal for all countries since it fully respects the ethical issues of donation; however, replacement donation might be regarded as ethically valuable and efficient in some cultures. In addition, some authors estimate that replacement donors are as safe as VNRD and less costly to health-care systems (16, 17).

In our opinion, an interesting but highly debatable strategy that can significantly alleviate the burden of transfusion-transmitted infections in endemic areas (i.e., Africa) is to establish financial contracts with “safe” donors (committed to safe behavior). However, such compensation would fall into the for-profit category according to the Nuffield Council on Bioethics (18), which raises two points: (a) The first is that this strategy is beneficial to patients in terms of supply and safety, as these donors ought to be at least as safe as or most probably safer than ordinary donors. (b) The second is related to ethical issues: are ethical values so inflexible, universal, and really independent from culture? This is perhaps debatable.

Emerging transfusion systems should also consider donor hemovigilance—set up over a decade after patient hemovigilance—especially when populations’ iron stores are threatened by many local reasons such as ethnicity, nutritional aspects, and digestive parasitosis. Furthermore, the frequency of donations also has a strong impact on donor safety and the depletion of iron stores. Finally, the ethics of donation or collection is now regarded as a strong pillar of safety (19).

Thus, it would be an interesting option for each country to consider the establishment of a Blood Supply Committee to discuss the organization and ethical issues of blood supply according to local characteristics and constraints. This committee should comprise not only of professionals and authorities but also representatives from various branches of human and social sciences (economy, ethics, sociology, etc.), and other stakeholders.

### BC Processing and Quality Management

The “B” leg of the transfusion stool encompasses the BCs and the quality management system. Nowadays, blood is collected, anticoagulated—which is a manipulation—and processed. Any BCs that are made available need to be defined (whole blood, red blood cell concentrates, plasma for therapeutic use, platelet components, etc.), along with their characteristics (volumes, active compounds with their minimum therapeutic levels, quality indicators, etc.). Furthermore, BCs can be either leukoreduced or not, and, if so, certain indicators must be defined such as date and time, pre- or post-storage (at bedside) leukoreduction and its efficacy, storage conditions (temperature), storage period, and expiry date. BC modifications such as irradiation, pathogen inactivation where available, volume reduction, washing, and splitting or pooling must be considered to define what is accepted by the system and what is not, and in which conditions. Quality indicators at all stages may be set up to monitor each part of the entire process (20).

Finally, testing of either the donor or the donated blood product is mandatory and not optional, but its extent and the decision tree upon biological findings may vary between systems. Indeed, no transfusion system can consider not testing donors for HIV, but not all HIV tests are equal nor are they confirmatory. Furthermore, some systems now retest previously donated BCs (using frozen plasma samples) in the event of positivity on the current donation.

However, not all systems allow the retrieval of historical samples. All of this depends on the organization, policy, and resources allocated to blood testing. An issue which is likely addressed in well-established systems is the definition of acceptance or rejection criteria for a given product (volume, quality, infectious safety level, extended blood grouping/phenotype, and residual leukocyte count to control or reduce inflammation). In fact, defining the infectious safety of a given BC (e.g., for a given virus) is a very difficult task. For example, is the residual risk of HIV-1 infection defined by less than 1 in 10^6^ donations, considered within the acceptable range? This may be unacceptable in countries that control HIV transmission well (i.e., in Europe or the USA), but unachievable in countries with a high prevalence of HIV in their population such as Middle Africa (21). It is clearly not the responsibility of HBB or Blood Establishment (BE) professionals to define such criteria, but rather the national public health authorities.

### Recipients (the Beneficiaries of BC Transfusions)

The “C” leg of the transfusion stool represents the recipients. Once the BC is qualified for issuing (and subsequently labeled), which is the main responsibility of the BE alongside the procurement of BCs that meet the demand, there remain a large number of tasks that the HBBs still have to perform, as they have to match the immunogenic and immunophenotypic characteristics of donor and recipient bloods. This is never a simple process in high-income countries and can be extremely difficult in low-income countries due to scarce resources: BCs in the inventory, resources to type and match bloods, etc. The financial burden of transfusions in high-income countries, where BC access was not restricted, has been extensive for decades with probable over-transfusion (22, 23). Those countries have now started to reduce unnecessary transfusions, implementing recommendations on Optimal Blood Use (OBU) and operating the so-called PBM programs (24, 25). Should low-income countries, where over-transfusion does not likely exist, start implementing PBM programs? And should they also apply OBU programs? We would be tempted to answer yes to both questions. In fact, both programs are aimed at improving the quality of medical services delivered to patients in need and reducing complications. Issuing recommendations that follow the general (universal) standards and are adapted to the actual situation of the country/system would be beneficial to both patients and the transfusion systems.

The major issue that developing countries face is the scarcity of surveillance and hemovigilance programs, with limited re-sources to recognize and report adverse reactions to implement improvement programs based on quality management. Another obstacle is the lack of evaluation of applicable or newly developed practices. Finally, hospital transfusion committees should be encouraged since they have proved to support transfusion safety in many places (26).

## Concluding Remarks

All of the foregoing raises the question of whether Eastern/Southern Mediterranean countries can either comply with Western countries’ guidelines and experiences or develop their own safety scheme based on proper sociopolitical and economic features. Another option (which does not necessarily contradict the previous ones) is to identify efficient and cost-effective strategies in neighboring countries that have had successful experiences and share similar cultural and economic features. To address this issue, and attempt to achieve this goal, we designed a number of surveys addressed specifically to Southern/Eastern Mediterranean countries that were sent out to national authorities when they existed, or to pre-identified blood banking specialists. So far, five surveys (comprising of 45 pages in total) have been produced and disseminated aimed at covering all aspects of blood activities [1—organization of the national transfusion service related to donors and staff; 2—prevention of infectious risks and prevalence of infectious diseases; 3—type, quantity, and specifications of produced blood products; 4—quality management system and the specifications of the environment of transfusion practices (education, vigilance, and invoicing); and 5—conditions of release and the use of blood and blood products] to collect and analyze data and standards. The surveys include series of questions tracking carefully all transfusion procedures that can be answered with a YES or NO and sometimes a box exists for some questions to place comments. All Southern/Eastern Mediterranean countries were targeted but only eight (Southern: Egypt, Morocco, Tunisia, Mauritania, and Algeria; Eastern: Lebanon, Jordan, and Palestine) responded to these surveys, which are currently being analyzed and validated before their communication. The preliminary results indicate that the organization of blood service in these countries is heterogeneous, as some countries have national systems and some others a decentralized organization; all countries nevertheless face similar challenges; to cite some: the blood supply relies mainly on replacement male donors; there is no clear strategy to secure the infectious safety of blood (and for instance no nuclear acid testing); there is not always an adequate quality management system; there is an evident lack of proper education; hemovigilance, when existing, is stammering, alongside all vigilances and surveillance processes. Some interesting experiences (e.g., universal leukoreduction in Lebanon, production of derived plasma products in Morocco, etc.) deserve to be highlighted and discussed regarding their outcomes and cost-effectiveness. It is anticipated that such a practice can help identify and then share the more successful and cost-effective experiences, and really focus on Mediterranean areas while not necessarily copying and pasting experiences designed for Western/Northern areas with significantly distinct situations in terms of donors, recipients, politics, economics, and even ethical and philosophical baseline.

## Author Contributions

AH and OG designed and wrote the paper. TA assisted with the writing and critical revision of the paper.

## Conflict of Interest Statement

The authors declare that the research was conducted in the absence of any commercial or financial relationships that could be construed as a potential conflict of interest.

The reviewer TB declared a past co-authorship with one of the authors OG to the handling Editor.
